# Supplementary material for: ProteinShader: illustrative rendering of macromolecules
Source: BMC Struct Biol. 2009 Mar 30;9:19. doi: 10.1186/1472-6807-9-19 (PMC2672931; doi:10.1186/1472-6807-9-19)
Supplement: Additional file 1 — ProteinShader program without source code. This compressed file contains the complete ProteinShader program including associated libraries, but no source code. A README.txt file gives an overview of the ProteinShader distribution, and the index.html file in the help subdirectory has directions on getting started with the program as well as a set of tutorials. [file 1472-6807-9-19-S1.zip › ProteinShader-beta-0_9_4-binary/help/tutorials/1JUN.html]

ProteinShader Tutorial: 1JUN


# ProteinShader Tutorial


♦ Home


♦ Download


♦ Getting Started

Choose a Link:

Installation

Running

Troubleshooting

PDB Files


♦ Tutorials

Choose a Link:

List of Proteins

Style Options

Saving Images


♦ General Controls

Choose a Link:

Menu Bar

Keyboard Shortcuts

Mouse Motion

Motion Panel

Antialiasing


♦ Cartoon Controls

Choose a Link:

Control Panels

Selection Panel

Radio Buttons

Decorations Panel

Adding Textures

Color Panel

Visibility Panel

Side Chains Panel


♦ Atom Controls

Choose a Link:

Control Panels

Selection Panel

Radio Buttons

Color Panel

Visibility Panel

Scale Panel

Tiling Panel


♦ Developers

Choose a Link:

Compiling

Running

Code Docs

Developer Links


## c-Jun Homodimer: Leucine Zipper Protein [PDB:1JUN]

The c-Jun protein is a transcriptional activator that binds DNA as either a
homodimer or a heterodimer with the c-Fos protein
[1, 2].
The three-dimensional structure of the leucine zipper domain of the c-Jun
homodimer has been determined by nuclear magnetic resonance imaging
[3, 4],
and can be loaded by selecting Open from the
File menu
of the ProteinShader program and then using the file chooser box
to select the 1JUN.pdb file.


## Cartoon and space filling style displays

When the c-Jun leucine zipper domain is first loaded, it will be displayed as a
pen-and-ink like drawing of ribbons (general loop regions) and tubes
(alpha-helices) as shown in Figure 1A below. The domain is composed of two
identical parallel chains, A and B, that are primarily alpha-helical in
structure. To switch to a display style that uses spheres to represent
atoms (Figure 1B), go to the
Style menu
above the canvas and use the Atom submenu to select Space Filling.
The default size of each sphere is determined by the van der Waals
radius for the atom type, which can be found by going to the
ProteinShader API and clicking on AtomEnum.
The default color is determined by the CPK (Corey, Pauling, and Koltun) color
scheme for atoms, which can be found by going to the
ProteinShader API and clicking on
CPKColorEnum.

With colors based on atom type, it is very difficult to distinguish the two
chains in a space filling style display, so in Figure 1B chain A is shown in red
and chain B in green. To set these colors, go to the menu at the top right
of the control panel and select
Atom Color.
Near the top left of the control panel, go to the Chain menu and select B.
Choosing B should cause the menu right below the Selected radio button to show
Chain as the current selection to apply modifications to. Clicking on the
Chooser button will open a color chooser panel, which can now be used to select
green for chain B. Leaving the color chooser panel open, go back to the
Chain menu and select A so that red can be applied to chain A.
Click on the OK button of the color chooser panel to close it.

To get a better idea of how the chains wrap around each other, either drag
the mouse horizontally across the canvas, or select
Motion
from the menu at the top right of the control panel and hit the Start button.
The text box or slider control below the Y-Axis label can be used to adjust
the speed of rotation. The motion panel sets speed in degrees per second
so that the rotation speed should be the same on any machine, but how smooth
or jerky the motion is will depend on how powerful the graphics card is.
After the image has been moved around, the original view can always be restored
by going to the
Orientation menu
above the canvas and selecting Original.


|  |  |
| --- | --- |
| **A** | **B** |

**Figure 1.** The leucine zipper domain of the c-Jun transcription factor
is a coiled coil of parallel alpha-helices. (A) The two parallel chains are
shown as ribbons and tubes using halftoning to produce a pen-and-ink style
drawing. (B) The same structure is shown as a space filling style display with
chain A in red and chain B in green. The images were captured as described in
the
Saving Images
section of the tutorials main page.


### Visualizing the leucine zipper

Like other members of the bZIP family of transcription factors, the c-Jun
homodimer is stabilized by hydrophobic interactions between leucine residues
that repeat at regular intervals along each chain
[2, 3].
To visualize these leucine residues, first go to the
Style menu above the
canvas and select Tubes from the Cartoon submenu. The loops will be shown with
a smaller diameter than the alpha-helix tubes. Now go to the menu
at the top right of the control panel and select
Cartoon Side Chains. Near the middle of the Cartoon Side Chains subpanel
is a radio button labeled Global. Go to the menu right below Global and
choose LEU. Now click the Space Filling button further below. The image
should be that same as is in Figure 2A below, with the leucine side chains
shown in red for chain A and green for chain B.

To get a better look at how the leucines pair up, rotate the image a few times
by dragging the mouse horizontally across the canvas. It may also be helpful
to temporarily set one of the chains to invisible as in Figure 2B, so go to the
menu at the top right of the canvas and select
Cartoon Visibility. Select either A or B from the Chain menu near the top
left of the control panel. Make sure that the menu right below the Selected
radio button is set to Chain, and then hit the Invisble button. With only a
single chain visible as in Figure 2B, it should be easy to observe that the
leucine residues are spaced every two turns of the alpha-helix, which
corresponds to every seventh residue.

To better observe the pairing between the hydrophobic side chains of the
leucine residues, the tubes used to represent the backbone of the alpha-helices
can be set to be partially transparent as in Figure 2C. Go to the menu right
below the Selected radio button to choose Model, and then click on the
Translucent button near the bottom of the Cartoon Visibility subpanel. The
translucent effect works a little better with a light background, so go to the
Background menu
above the canvas and select Light Gray. The slider
near the bottom of the Cartoon Visibility panel can be used to fade the
translucent tubes in and out. The side chains are not affected because they
are an atom-style display, so the Atom Visibility subpanel would affect their
translucency, not the Cartoon Visibility subpanel.


|  |  |  |
| --- | --- | --- |
| **A** | **B** | **C** |

**Figure 2.** Illustration of the leucine residues of the leucine zipper
domain of the c-Jun protein. (A) The two identical chains of the c-Jun
homodimer are shown as tubes, while the leucine side chains are shown as a space
filling display. The leucine residues for chain A are shown in red, while the
leucine residues for chain B are shown in green. (B) Chain A has been set to
invisible so that the leucine residues of chain B can be seen more clearly.
Leucine residues occur every two turns of the alpha-helix (every 7 residues).
(C) Same display as in (A), except that the tubes are shown using 75%
translucency to make it easier to see the pairing of the hydrophobic leucine
residues. The images were captured as described in the
Saving Images
section of the tutorials main page.


### Destabilizing the leucine zipper

In addition to the leucines that form a zipper-like structure, a particularly
interesting residue discussed by the authors of the c-Jun NMR paper is an
asparagine at position 291 of each chain [3].
This polar residue is located at the dimer interface, and provides a
destabilizing influence that is likely to facilitate the rapid exchange of
zipper strands *in vivo* (c-Jun/c-Fos heterodimers have a higher DNA
binding affinity than c-Jun homodimers, and control of dimer formation is part
of how this family of transcription factors is regulated).

To visualize this residue, first go to the list of amino acids shown on the
left side of the control panel and select ASN 291. Then use the menu at the
top right of the control panel to go back to the Cartoon Side Chain subpanel.
Make sure that Residues is showing in the menu below the Selected radio button,
and then click on the Space Filling button. To change the color of the
aspargine side chain, change the menu at the top right of the control panel to
Atom color and click the Atom Type button. Setting the color by atom type will
use gray for carbon, white for hydrogen, blue for nitrogen, and red for oxygen.

The manipulations described right above will affect only the ASN 291 of
whichever chain (A or B) is currently showing in the Chain menu near the top
left of the control panel, so the process will need to be repeated after
flipping the Chain menu to the other chain. Using the Chain menu will
cause the menu below the Selected radio button to automatically flip to
Chain, so it will be necessary to change that menu back to Residues before
clicking the Space Filling button on the Cartoon Side Chains subpanel or the
Atom Type button on the Atom Color subpanel. Once ASN 291 has been set on
both chains, the image should look the same as in Figure 3A below.
To get a better look at the asparagine residues, the
mouse motion controls
can be used to zoom in on the image as shown in Figure 3B.


|  |  |
| --- | --- |
| **A** | **B** |

**Figure 3.** A polar asparagine residue has a destabilizing influence on
the leucine zipper domain of the c-Jun homodimer. (A) The protein is shown
as a tubes style display (75% transparency) with leucine side chains (red for
chain A and green for chain B) as a space filling style display. The side chain
for asparagine 291 of each chain is also shown, but colored by atom type (gray
for carbon, white for hydrogen, blue for nitrogen, and red for oxygen). (B) The
same display as in (A) after zooming in on the aspargine residues and rotating
the image. The images were captured as described in the
Saving Images
section of the tutorials main page.


### Multiple models

As a final point for this tutorial using c-Jun, there are actually seven
slightly different models for the leucine zipper domain because the structure
was determined using NMR (nuclear magnetic resonance imaging), which only
provides a set of distance constraints for hydrogen atoms
[5].
The ProteinShader program loads all of the models given in a Protein Data
Bank file, and the Model menu at the top left of the control panel can be used
to select any of the models. Currently, the program makes no attempt to
synchronize changes to the models, but that option might be added at a later
time. Right now, when a structure is first loaded, the
Decorations subpanel
is used to automatically apply Halftoning with texture maps (to get a
pen-and-ink style effect) to only the first model. To apply the same effect to
other models, the Halftoning button and its associated texture menus should be
used. Any modifications to one model are remembered by only that one model.


## References

1.
Brandon C, Tooze J: **Leucine zippers provide dimerization
interactions for some eucaryotic transcription factors.**
In *Introduction to Protein Structure.* 2nd edition.
New York: Garland Publishing; 1998: 191-193.

2.
Angel PE, Herrlich PA (Eds): *The FOS and JUN Families of Transcription
Factors.* Boca Raton: CRC Press; 1994.

3.
Junius FK, O`Donoghue SI, Nilges M, Weiss AS, King GF:
**High resolution NMR solution structure of the leucine zipper
domain of the c-Jun homodimer.**
*J Biol Chem* 1996, **271**: 13663-13667.

4.
**c-Jun homodimer PDB entry 1JUN**
[http://www.rcsb.org/pdb/explore/explore.do?structureId=1JUN]

5.
Brandon C, Tooze J: **Determination of Protein Structure.**
In *Introduction to Protein Structure.* 2nd edition.
New York: Garland Publishing; 1998: 373-392.


---

|  |  |  |  |
| --- | --- | --- | --- |
| Hosted by |  |  |  |

Webmaster: Joe Weber,
joe.weber AT alumni.duke.edu
  
Web site last updated: December 30th, 2008
  
© 2007–2008 Joseph R. Weber
